# The Role of RASSF1C in the Tumor Microenvironment

**DOI:** 10.3390/cimb45020074

**Published:** 2023-01-31

**Authors:** Yousef G. Amaar, Mark E. Reeves

**Affiliations:** 1 Surgical Oncology Laboratory, Loma Linda VA Medical Center, Loma Linda, CA 92357, USA; 2 Department of Surgery, Loma Linda University, Loma Linda, CA 92350, USA

**Keywords:** cell migration, metastasis, RASSF1C, PIWIL1, P4HA2, PLOD2, piRNAs, gene expression

## Abstract

The tumor microenvironment (TME) plays a vital role in tumor invasion and metastasis and provides a rich environment for identifying novel therapeutic targets. The TME landscape consists of an extracellular matrix (ECM) and stromal cells. ECM is a major component of TME that mediates the interaction between cancer cells and stromal cells to promote invasion and metastasis. We have shown in published work that RASSF1C promotes cancer stem cell development, migration, and drug resistance, in part, by promoting EMT through a mechanism that involves up-regulation of the PIWIL1-piRNA axis. Consistent with this, in this study, we demonstrate that RASSF1C promotes lung cancer metastasis in vivo using an orthotopic mouse model. Interestingly, two target genes identified in a previously conducted microarray study to be up-regulated by RASSF1C in breast and non-small cell lung cancer (NSCLC) cells are prolyl 4-hydroxylase alpha-2 (P4HA2) and procollagen-lysine, 2-oxoglutarate 5-dioxygenase 2 (PLOD2). In cancer, P4H2A and PLOD2 are vital for collagen posttranslational modification and folding leading to the formation of a stiff ECM and induction of EMT and cancer stem cell marker gene expression, resulting in metastatic dissemination. Here, we also show that overexpression of RASSF1C up-regulates Collagen I, P4HA2, and PLOD2 in vitro. Up-regulation of P4HA2 and PLOD2 by RASSF1C was also confirmed in lung and breast cancer cells in vivo using mouse models. Further, we found that treatment of wildtype lung cancer cells or lung cancer cells overexpressing RASSF1C or PIWIL1 with piR-35127 and 46545 (both down-regulated by RASSF1C) decreased lung cancer cell invasion/migration. Taken together, our findings suggest that RASSF1C may promote lung cancer cell ECM remodeling to induce lung cancer cell stemness, invasion, and metastasis, in part, by up-regulating a previously unknown PIWIL1-P4HA2-PLOD2 pathway. Furthermore, piR-35127 and piR-46545 could potentially be important anti-metastatic tools.

## 1. Introduction

Lung cancer is the leading cause of cancer deaths in the United States, exceeding that of all other cancers combined [1]. The heterogeneity of this disease has dictated the development of “precision” medicine approaches to improve lung cancer treatment. Novel treatment regimens are now possible with drugs that target mutated driver genes and their downstream pathways [2,3]. Thus, patients will most likely require a combination of precision drugs together with chemotherapy and radiation. Therefore, there is a need to identify additional driver pathways and their downstream gene networks that can be targeted to effectively treat late-stage and metastatic lung cancer. 

Our laboratory focuses on the oncogenic activities of the Ras Association Domain Family Member 1 (RASSF1) gene in breast and lung cancer. RASSF1 encodes two major isoforms, RASSF1A and RASSF1C, derived by alternative promoter selection and mRNA splicing [4,5,6]. RASSF1A is a tumor suppressor [4,5,6,7,8,9], whereas RASSF1C appears to function as an oncogene [10,11,12,13,14,15,16,17]. Our previous studies show that a significant fraction (>50%) of lung cancers are characterized by elevated RASSF1C or RASSF1C/RASSF1A ratios [12]. We have also demonstrated that RASSF1C stimulates in vitro cell cycle, proliferation, and migration of human breast and lung cancer cells [10]. Consistent with this, we found that RASSF1C regulates the expression of several genes/proteins important in maintaining a CSC-like phenotype and oncogenesis [11,15]. We have shown that RASSF1C induces expression of PIWIL1 and accumulation of β-catenin (both associated with stem cell self-renewal) and regulates expression of PIWI-interacting RNAs (piRNAs) associated with stem cell function in lung cancer cells [11,14]. In addition, we found that modulation of RASSF1C and PIWIL1 gene expression alters DNA methylation of specific oncogenes and tumor suppressors in lung cancer cells suggesting that the RASSF1C-PIWIL1-piRNA pathway could influence epigenetic modifications to drive cancer cell progression and metastasis [18]. Further, among the piRNAs that are negatively regulated by RASSF1C, we found that overexpression of piR-35127 and piR-46545 decreased lung cancer and primary epithelial cell proliferation and colony formation [16]. We also should note that small molecules that induce (e.g., Dorsomorphin, AMPK inhibitor) or attenuate RASSF1C expression (ERK inhibitor, CI-1040 and AMPK activator, Trichostatin A) have corresponding effects on PIWIL1 and piRNA gene expression [14,16]. The objective of this study was to further our understanding of the oncogenic activities of the RASSF1C-PIWIL1 gene axis in lung cancer cells related to growth and metastasis, along with the underlying mechanism(s).

It has been well documented that the tumor microenvironment (TME) plays a vital role in tumor invasion and metastasis as it provides a rich environment for identifying novel therapeutic targets [19,20]. The TME landscape consists of ECM, vasculature, cancer-associated fibroblasts, and infiltrating immune cells [19,20,21,22]. ECM is a major component of TME that mediates the interaction between cancer cells and stromal cells to promote invasion and metastasis. The collagens, proteoglycans, and glycosaminoglycans in ECM can create a stiff, dense environment that promotes cancer stem cell programming associated with cancer invasion, migration, and metastasis [19,20,21,22,23,24]. In this regard, we report that RASSF1C and PIWIL1 may promote lung cancer cell metastasis through modulation of tumor microenvironment/ECM through regulation of Collagen I, P4HA2, and PLOD2 gene expression. We also report that piR-46546 and piR-35137 attenuate invasion/migration of lung cancer cells overexpressing RASSF1C or PIWIL1 in vitro. 

## 2. Materials and Methods

### 2.1. Cell Culture

Lung cancer cell lines H1299 and A549 were used in this study. H1299 cells were stably transduced with either GFP, GFP-1C, or GFP-PIWIL1 using lentiviral particles (Origene, Rockville, MD, USA) using a standard protocol. A549 overexpression backbone (A549-BB) or expressing RASSF1C (A549-1C) were prepared as previously described [14,15]. Cells were cultured in the appropriate medium supplemented with 10% calf bovine serum as previously described [14,15]. 

### 2.2. Lung Metastasis Model

An orthotopic mouse xenograft model of lung cancer metastasis [19] was used to demonstrate that RASSF1C promotes lung cancer cell metastasis in vivo. In this model, 0.5 × 10^6^ human A549-BB, A549, 1C, H1299-GFP, H1299-GFP-1C were injected orthotopically in the left lung parenchyma of 6-week-old athymic nude mice (NCr nu/nu; Taconic, Germantown, NY, *n* = 4) from which they metastasize to right lung. Eight weeks post cell injections, animals exhibited right lung metastasis. Lungs were collected and processed for histology work using standard protocols. The animal study protocols were approved by the Loma Linda VA Medical Center IACUC.

### 2.3. Subcutaneous Animal Model

We used T47D xenograft sections from paraffin-embedded blocks, from a previous study, derived from T47D cells overexpressing HA-RASSF1A, HA-RASSF1C, or T47D-vector backbone [13]. 

### 2.4. Histology and Immunohistochemistry

Immunohistochemical stainings of xenograft sections derived from the orthotopic and subcutaneous mouse models were carried out with the diaminobenzidene (DAB) method. Five-micrometer sections cut from paraffin-embedded blocks were deparaffinized. Antigen retrieval was performed using Sodium Citrate buffer (10 mM Sodium Citrate, 0.05% Tween 20, pH 6.0). Slides were immersed in Sodium Citrate buffer and incubated at 95–100 °C for 20–40 min and then slides were cooled to room temperature before washing them 2X with 1XPBS + 0.05% Tween 20. Slides were incubated in 3% H_2_O_2_ for 30 min at room temperature. Sections were then washed 3X with 1XPBS + 0.05% Tween 20 and blocked with 5% normal goat serum (Vector Laboratories) overnight at 4 °C. Goat serum was removed and 1:100 dilution of rabbit primary antibody was applied overnight at 4 °C. Sections were washed three times for 5 min each with 1XPBS/Tween, and 1:1000 horseradish peroxidase secondary (Vector Laboratories) was then applied for 60min at room temperature. Sections were washed three times with PBS/Tween and DAB chromogen (Biocare Betazoid DAB Chromogen, Invitrogen, Waltham, MA, USA) for 5 min at room temperature and sections were washed 3X with H_2_O and counterstained with Hematoxylin (Harris Hematoxylin, Fisher Scientific, Waltham, MA, USA).

### 2.5. RT-PCR Analysis

Total RNA from human lung cancer cells was isolated and (RT)-PCR was performed using gene-specific primers as previously described [6]. PCR was carried out using HotStart and Sybergreen master mixes (Qiagen, Valencia, CA, USA). The RT-PCR reactions were carried out in triplicates and the fold change was calculated using the 2^−ΔΔCT^ method. P4HA2 and PLOD2 RT-PCR analysis was carried out using specific gene primers and Cyclophilin gene expression (internal control) was assessed using gene-specific primers. P4HA2-F: ATGTAGAAGCTGGTGGTGC; P4HA2-R: TTGACATGGGCTGAAGGACCS; PLOD2-F: CACCGACGACCTCACTCAG; PLOD2-R: TTCTGGCCCCCTCCAATACT; Cyclophilin-F: GCATACAGGTCCTGGCATCT; and Cyclophilin-R: GCTCTCCTGAGCTACAGAAG.

### 2.6. Cell Migration Assay

H1299 cells were plated at 2 × 10^4^ cells per chamber and the next day were transfected with GFP or GFP-RASSF1C or GFP-PIWIL1 lentiviral vectors. Cells transfected with MOI of 5 and pure clones were selected using GFP-expression. For the Bowden chamber assay, a cell was plated at 25,000 per chamber and the next day cells were transfected with piRNAs (piR-35127 and piR-46545) at a final concentration of 1 uM using Lipofectamine 2000. Cells were processed 24–48 h post-transfection and were fixed with methanol for 2 min and stained with 1% Toluidine blue for 2 min as previously described [14,15]. The stained Bowden chambers were examined under a bright field microscope and were photographed and colonies were counted. 

### 2.7. Western Blot Analysis

Immunoblots were performed using gene-specific antibodies. Western blot analysis of experimental and control cell lysates was carried out using the Odyssey^®^ Infrared System (LI-COR Biosciences, Lincoln, NE, USA). Cell lysate from control and experimental cells was prepared using RIPA lysis buffer supplemented with 1X protease inhibitors (Sigma, St. Louis, MO, USA) and 25 µg of cell lysates was used to run Western blots. P4HA2 (Cat #HPA027824) antibody was purchased from Sigma Inc (Sigma, St. Louis, MO, USA). PLOD2 (Cat # 50-557-311) was purchased from Fisher Scientific (Fisher Scientific, Pittsburgh, PA, USA). Collagen I (Cat # 1310-01) antibody was purchased from Southern Biotech (Birmingham, AL, USA). Polyclonal beta actin antibody (Cat # sc-1615) was purchased from Santa Cruz Biotechnology, Inc. (Santa Cruz, CA, USA), and fluorescently labeled secondary antibodies IRDye^®^ 680 and 780 RD Infrared Dye were purchased from LI-COR (LI-COR Biosciences, Lincoln, NE, USA). The experiments were repeated at least 3 times. Protein levels were normalized to actin levels (loading control). The average intensities of signals on Western blot images were quantified and normalized using the Odyssey image analysis software.

### 2.8. Immunofluorescence Analysis

Immunofluorescence was carried out using gene-specific antibodies and Alexa fluor secondary antibodies. Cells were fixed in 4% paraformaldehyde for 10 min and rinsed with 1XPBS three times before cells were permeabilized with 1XPBS + 0.05% triton x100 for 10 min. Cells were washed 3X with 1XPBS + 0.05% Tween 20 before incubating cells with blocking buffer in 2.5% rabbit serum in 1XPBS + 0.05% Tween 20 overnight at 4 °C before incubating with rabbit primary antibodies overnight at 4 °C. After incubation with primary antibodies, cells were washed 3X with 1XPBS + 0.05% Tween 20 and incubated with Alexa flour secondary antibodies for 1 h. Cells were subsequently washed 3X and were imaged using fluorescence microscopy.

### 2.9. Statistical Analysis

The *t*-test was used to calculate the significance of migration and cell proliferation data. All experiments were run at least three times and data were used to perform statistical analysis.

## 3. Results

### 3.1. RASSF1C Overexpression Promotes Lung Cancer Cell Metastasis In Vivo

**Figure 1:**
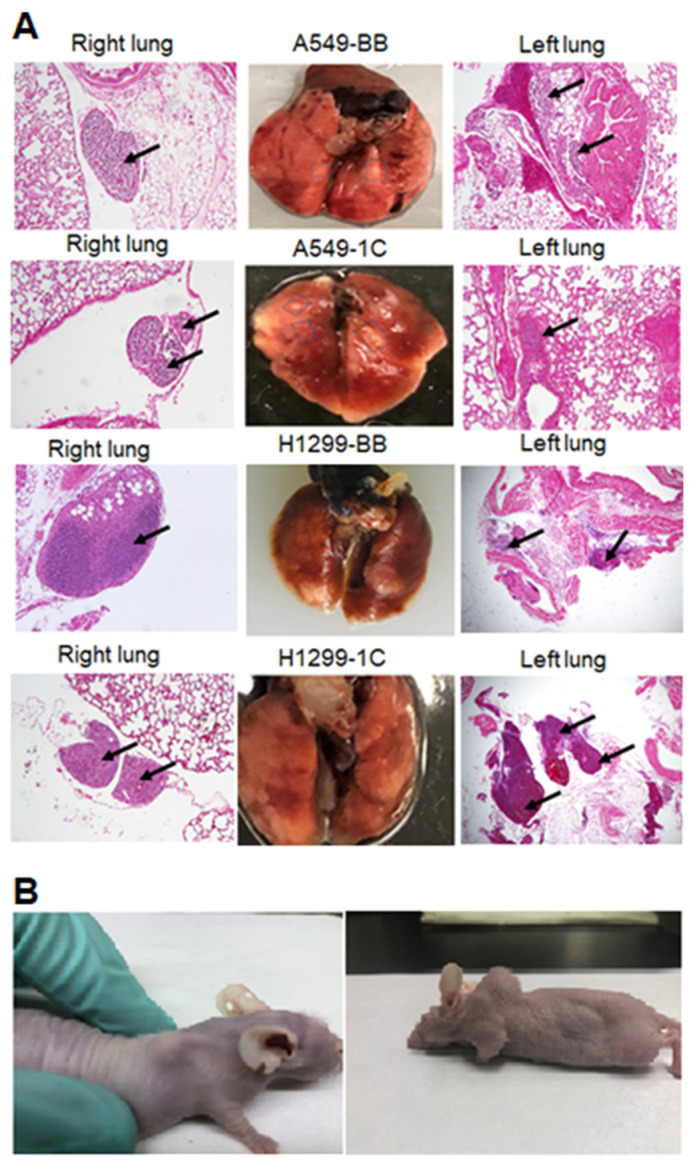
RASSF1C promotes lung cancer cell metastasis in vivo. Lung cancer cell lines A549 ((**A**)-upper panel) and H1299 ((**A**)-lower panel) overexpressing vector backbone (BB) or RASSF1C were orthotopically injected in the left lung of nude mice (*n* = 3). Eight weeks post cell injections, animals exhibited right lung metastasis. Some animals exhibited subcutaneous metastases as well (**B**). Image magnification is at 4×.

We previously showed that RASSF1C promotes lung cancer cell migration in vitro [14,15]. To validate that the overexpressing of RASSF1C in lung cancer cells, unlike RASSF1A, promotes lung cancer cell metastasis, we used an orthotopic mouse xenograft model of lung cancer metastasis [19]. We demonstrate that injecting 0.5 × 10^6^ human NSCLC cells (NCI-H1299 or A549) overexpressing RASSF1C or vector backbone in the left lung parenchyma of 6-week-old athymic nude mice metastasize to the right lung and distant organs (Figure 1). Our results clearly demonstrate that RASSF1C promotes lung cancer cell metastasis in vivo, further supporting our previously published in vitro findings, and clearly demonstrate that RASSF1C acts as a pro-oncogenic factor promoting lung tumor growth, survival, and metastasis.

### 3.2. RASSF1C Overexpression Up-Regulates P4HA2 and PLOD2 Gene Expression In Vitro

**Figure 2:**
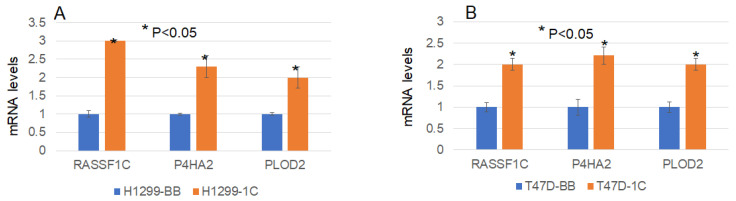
RASSF1C overexpression up-regulates expression of P4HA2 and PLOD2 in lung cancer cell line H1299 (**A**) and breast cancer cell line T47D (**B**) as shown by RT-PCR analysis. The fold change was calculated using the 2^−ΔΔCT^ method [25].

**Figure 3:**
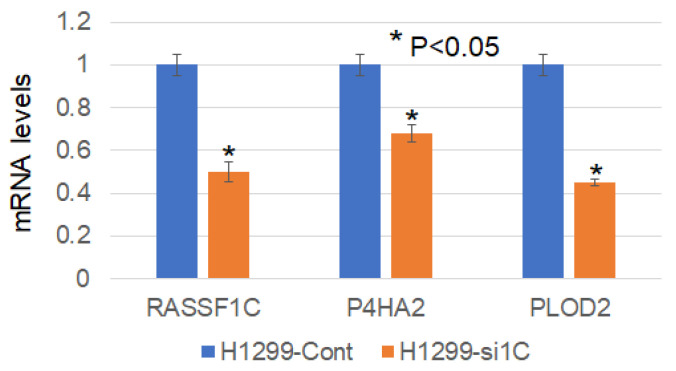
Knockdown of RASSF1C (1C-KD) expression decreased P4HA2 and PLOD2 mRNA levels in lung cancer line H1299 as shown by RT-PCR analysis. The fold change was calculated using the 2^−ΔΔCT^ method [25].

**Figure 4:**
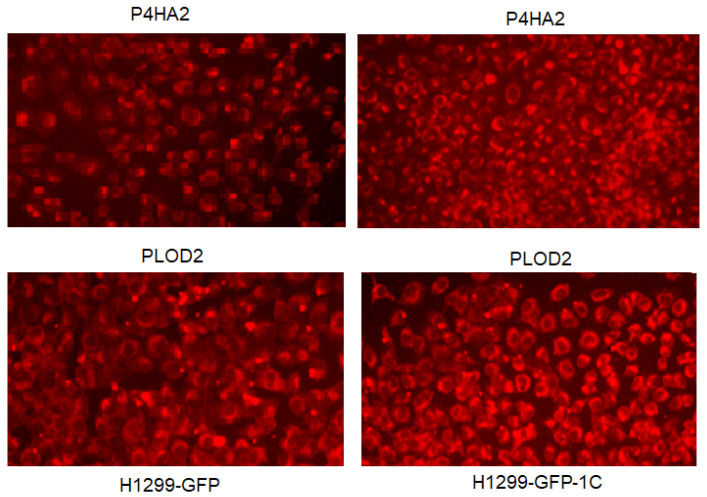
Immunofluorescence staining of P4HA2 and PLOD2 protein in H1299 expressing GFP or GFP-RASSF1C (H1299-GFP-1C). Primary antibodies: rabbit anti-P4HA2 and PLOD2; secondary antibody: goat-anti-rabbit Alexa flour 546. Images magnification at 10×.

**Figure 5:**
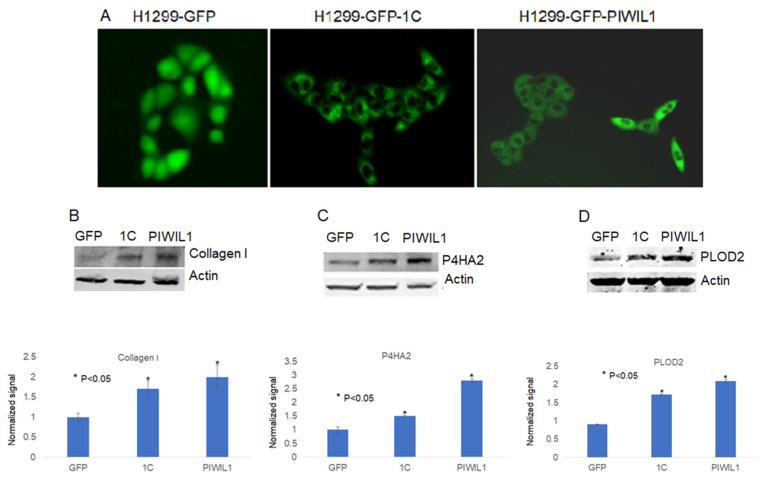
Assessment of P4HA2, PLOD2, and collagen I protein levels in the lung cancer cell line H1299 stably expressing GFP, expressing GFP-RASSF1C (1C), or GFP-PIWIL1 (**A**). Blots were probed with Collagen I (**B**), P4HA2 (**C**), and PLOD2 (**D**). Cells overexpressing GFP-RASSF1C display higher Collagen, P4HA2, and PLOD2 protein levels compared to the control (GFP). Actin was used as a loading control.

**Figure 6:**
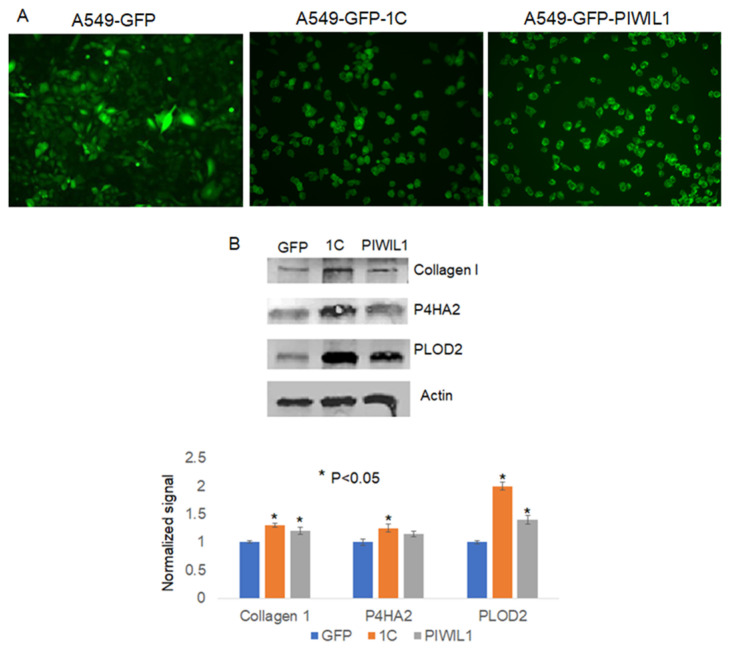
Assessment of P4HA2, PLOD2, and collagen I protein levels in the lung cancer cell line A549 stably expressing GFP, expressing GFP-1C (1C), or expressing GFP-PIWIL1 (PIWIL1) (**A**). (**B**) Western blot was probed with Collagen I-, P4HA2-, and PLOD2-specific antibodies. Cells overexpressing GFP-RASSF1C or GFP-PIWIL1 display higher P4HA2, PLOD2, and collagen 1 protein levels compared to control (GFP).

To further shed some light on how RASSF1C may promote metastasis, we searched RASSF1C target genes we previously identified in breast and lung cancer cells [11,15] using microarray studies. We found that P4HA2 and PLOD2 were up-regulated in both breast and lung cancer cell lines, T47D and H1299, respectively (Table 1). P4HA2 and PLOD2 expression was confirmed in breast and lung cancer cells overexpressing RASSF1C by RT-PCR (Figure 2) [25]. Further, we assessed P4HA2 and PLOD2 expression in H1299 cells with silenced RASSF1C expression (Figure 3) and found that the expression of P4HA2 and PLOD2 was down-regulated. Consistent with RT-PCR data, immunofluorescence (Figure 4) and immunoblotting (Figure 5 and Figure 6) show increased levels of P4HA2 or PLOD2 protein levels in cells overexpressing RASSF1C. In addition, increased collagen I protein levels in H1299 and A549 cells overexpressing RASSF1C were detected (Figure 5 and Figure 6). Our findings suggest a potential mechanism involving RASSF1C to promote breast and lung TME remodeling that is perhaps HIF1-independent as our data are obtained under non-hypoxic conditions.

### 3.3. RASSF1C Overexpression Up-Regulates P4HA2 and PLOD2 Gene Expression In Vivo

**Figure 7:**
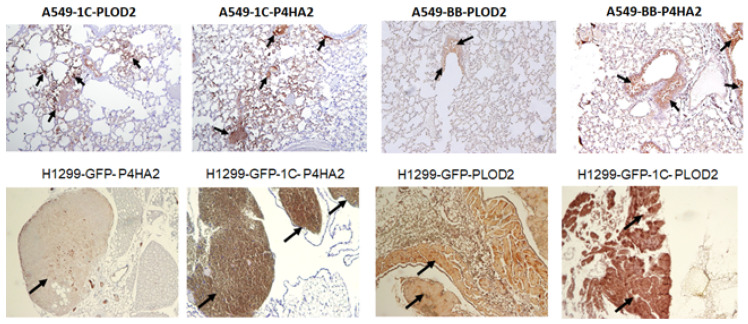
Immunohistochemical analysis was carried out of P4HA2 and PLOD2 in A549 and H1299 overexpressing RASSF1C and corresponding controls representing lung metastatic tumor nodules. The diaminobenzidene (DAB) method was used along with P4HA2 and PLOD2 primary antibodies to perform the staining. A549--1C and H1299-GFP-1C show higher levels of P4HA2 and PLOD2 expression compared to controls, A549-BB and H1299-GFP. Sections stained were prepared from right lungs showing metastatic nodules. Arrows indicated nodules positive for P4HA2 and PLOD2 stained with DAB. Image magnification is 10×.

**Figure 8:**
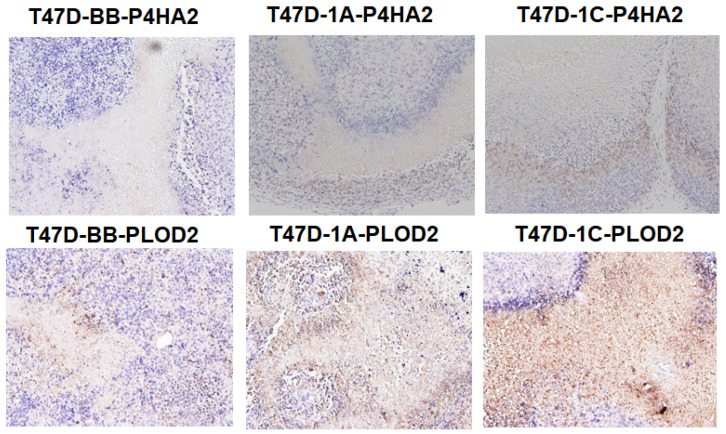
Immunohistochemical analysis was carried out for HA-RASSF1A and HA-RASSF1C fusion proteins in T47D derived from subcutaneous breast tumor sections [13] The DAB method along with P4HA2 and PLOD2 primary antibodies was used to perform the staining. T47D-1C tumor sections display higher P4HA2 and PLOD2 expression compared to T47D-1A and T47D-BB tumor sections. Images magnification is 10×.

We have assessed P4HA2 and PLOD2 expression in vivo using lung tissue derived from an orthotopic metastasis model using lung cancer cell lines A549 and H1299. Lung metastatic nodules derived from A549-HA-RASSF1C or H1288-GFP-RASSF1C show higher expression of P4HA2 and PLOD2 compared to A549-backbone or H1299-GFP controls (Figure 7). We also assessed the expression of P4HA2 and PLOD2 in breast xenografts derived from T47D cells overexpressing HA-RASSF1A, HA-RASSF1C, or vector backbone as previously reported [11,15]. We found that T47D-HA-RASSF1C xenografts displayed higher, while T47D-HA-RASSF1A xenografts displayed lower, P4HA2 and PLOD2 expression compared to T47D-vector backbone (Figure 8) suggesting that RASSF1C and RASSF1A have opposite effects on P4HA2 and PLOD2 gene expression. Taken together, our in vivo data are consistent with our in vitro data and suggest a role for RASSF1C in tumor microenvironment remodeling. P4H2A and PLOD2 are vital for collagen posttranslational modification and remodeling of the tumor microenvironment and ECM leading to the formation of a stiff ECM and induction of cancer cell stemness and metastatic dissemination [20,21,22,23,24,26].

Thus, our findings suggest that the RASSF1C-PIWIL1 pathway could potentially promote lung cancer microenvironment/ECM remodeling to induce lung cancer cell stemness and metastasis, in part, via modulation of P4HA2 and PLOD2 expression. 

### 3.4. PIWIL1 Promotes piRNAs Attenuate Lung Cancer Cell Invasion/Migration

**Figure 9:**
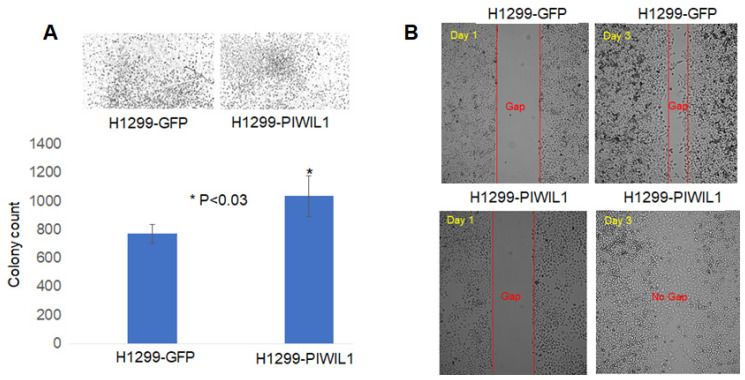
PIWIL1 promotes lung cancer cell migration. (**A**) Cell invasion assay. The BD BioCoatTM MatrigelTM Invasion Chamber was used to assess cell invasion/migration of metastatic lung cancer cell line NCI-H1299 overexpressing PIWIL1 (H1299-PIWIL1) or GFP (H1299-GFP). Overexpression of PIWI significantly increased the number of cells invading the Matrigel chamber and migrating to the other side of the filter vs. GFP control 48 h post-plating. (**B**) Wound healing assay shows H1299-PIWIL1 cells closed a wound gap at day 3 compared to H1299-GFP cells (*n* = 3). *t*-test at *p* < 0.05.

**Figure 10:**
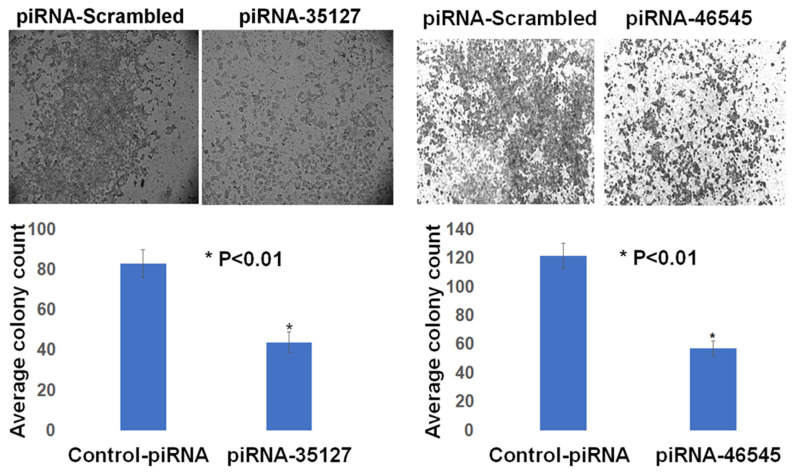
piRNA-35127 and piR-46545 attenuate lung cancer cell migration. The BD BioCoatTM MatrigelTM Invasion Chamber assay was used to assess cell invasion/migration of the lung cancer cell line NCI-H1299. Treatment of H1299 with 500 nM piR-35127 or piR-46545 mimics significantly decreased cells invading the Matrigel chamber and migrating to the other side of the filter vs. control 48 h post-treatment (*n* = 3). *t*-test at *p* < 0.05.

**Figure 11:**
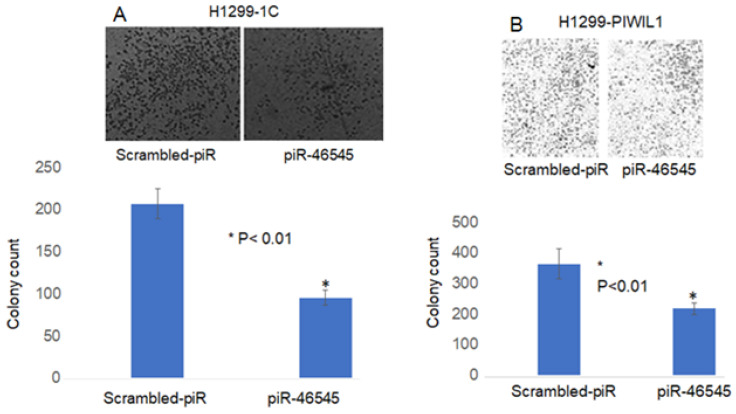
piR-46545 attenuates RASSF1C and PIWIL1 effects on lung cancer cell migration. Treatment of H1299-GFP-1C (**A**) and H1299-GFP-PIWIL1 (**B**) with 500 nM piR-46545 mimics significantly decreased cells invading the Matrigel chamber and migrating to the other side of the filter vs. control 48 h post-treatment (*n* = 3). *t*-test at *p* < 0.05.

In previously published work we reported that RASSF1C up-regulates PIWIL1 and down-regulates piR-35127 and piR-46545 gene expression in lung cancer cells and restoring expression of these two specific piRNAs in breast and lung cancer cells decreased lung cancer cell and primary epithelial cell proliferation and colony formation [16]. We found overexpressing PIWIL1 (Figure 9), like RASSF1C, promotes lung cancer cell migration/invasion and that PIWIL1 overexpression resulted in increased expression of P4HA2, PLOD2, and collagen I protein levels (Figure 5 and Figure 6) suggesting that PIWIL1 could play a role TME remodeling. In light of these findings, we assessed the impact of over-exprssing of piR-35127 and piR-46545 on lung cancer cell migration/inavsion and found that they drastically reduce wildtype lung cancer cell invasion (Figure 10) as well as lung cancer cells overexpressing RASSF1C or PIWIL1 (Figure 11). Together, our findings further support that piR-35127 and piR-46545 may function as tumor suppressors in lung cells, and down-regulation of piR-35127 and piR-46545 may contribute to lung cell transformation and tumorigenesis.

## 4. Discussion

We have demonstrated in previously published work that RASSF1C acts as an oncogene in both breast and lung cancer cells [10,11,12,13,14,15]. Consistent with our published work, we now demonstrate that RASSF1C overexpression, unlike RASSF1A, promotes lung cancer metastasis in an orthotopic mouse model as shown in Figure 1. H1299 or A549 lung cancer cells overexpressing RASSF1C injected in the left lung were able to metastasize to the right lung and in some animals resulted in the development of subcutaneous metastases. Our previously published work also shows that RASSF1C promotes EMT and lung cancer stemness [17], two events that are impacted by tumor microenvironment (TME)/extracellular matrix remodeling (ECM). We, therefore, wondered if RASSF1C could promote lung cancer cell metastasis, in part, through modulation of the important gene(s) involved in TME/ECM remodeling. To answer this question, we re-analyzed our microarray data of RASSF1C gene targets identified in breast and lung cancer cells conducted in previously published studies [11,15]. We were able to identify the P4HA2 and PLOD2 genes as RASSF1C target genes in both breast and lung cancer cells (Table 1). RASSF1C up-regulation of P4HA2 and PLOD2 expression in both breast and lung cancer cells was confirmed by RT-PCR analysis (Figure 2). We also found that silencing of RASSF1C in lung cancer cells resulted in the down-regulation of P4HA2 and PLOD2 (Figure 3). Consistent with RT-PCR analysis, lung cancer cells overexpressing RASSF1C had increased P4HA2, PLOD2, and collagen I protein levels (Figure 4 and Figure 5). Further, RASSF1C up-regulation of P4HA2 and PLOD2 expression was confirmed in vivo in lung cancer tissue developed in an orthotopic mouse model (Figure 7) and in breast subcutaneous tumor tissue (Figure 8) derived from breast cancer cells overexpressing RASSF1C or RASSF1A [13]. Thus, our in vitro and in vivo findings suggest that RASSF1C could play a role in promoting tumor microenvironment (TME) remodeling perhaps, in part, through collagen protein stabilization, hydroxylation, collagen fiber organization, and extracellular matrix (ECM) stiffness. TME plays a vital role in tumor invasion and metastasis [19,20,21,22,23] and ECM is a major component of TME that mediates the interaction between cancer cells and stromal cells to promote invasion and metastasis [24]. P4HA2 and PLOD2 are regulated by FOXA1, tumor growth factor-β (TGF-β), and hypoxia-inducible factor-α (HIF-1 α) to induce remodeling of extracellular matrix (ECM) and cancer cell stemness to drive tumor cell invasion and metastasis [26,27,28,29]. We should note that restoring RASSF1A expression in lung cancer cells activates the Hippo pathway leading to the inhibition of YAP-mediated transcription of P4HA2, cell stemness, ECM stiffness, and metastasis [24]. We have in previously published work highlighted the opposing effects of RASSF1A and RASSF1C on cell proliferation, migration, and apoptosis [12]. We also have shown that breast cancer cells overexpressing RASSF1C result in larger subcutaneous tumors compared to cells overexpressing RASSF1A [13]. Consistent with this, our IHC analysis of breast subcutaneous tumor tissues shows the expression of P4HA2 and PLOD2 in tissue derived from cells overexpressing RASSF1A is less pronounced compared to tissues derived from cells overexpressing RASSF1C and the vector backbone (Figure 8). This provides additional evidence to support the idea that RASSF1C and RASSF1A have opposite effects on breast and lung cancer cell growth and metastasis.

We have previously shown that RASSF1C induces expression of PIWIL1 and accumulation of β-catenin (both associated with stem cell self-renewal) and regulates expression of PIWI-interacting RNAs (piRNAs) associated with stem cell function in lung cancer cells [11,14]. In addition, we found that modulation of RASSF1C and PIWIL1 gene expression alters DNA methylation of specific oncogenes and tumor suppressors in lung cancer cells, suggesting that the RASSF1C-PIWIL1-piRNA pathway could influence epigenetic modifications to drive cancer cell progression and metastasis [18]. Recently published work showed that collagen hydroxylation promotes EMT in A549 lung cancer cells through DNA and histone demethylation/hydroxylation, and knockdown of P4HA2 in A549 lung cancer cell lines resulted in down-regulation of H3K9me2 and H3k36me3 and decreased cell migration [29]. Thus, one potential mechanism through which RASSF1C could potentially promote lung cancer metastasis is through regulation of the PIWIL1-piRNA axis. Hence, the RASSF1C-PIWIL1-piRNA pathway may present a novel mechanism involved in the regulation of P4HA2 and PLOD2 expression to impact TME remodeling. In support of this idea, we found that overexpression of PIWIL1 promotes lung cancer cell migration/invasion (Figure 9), which is consistent with published reports [30]; and cells overexpressing PIWIL1 display increased protein levels of P4HA2, PLOD2, and collagen I to some extent (Figure 5 and Figure 6). Treatment of wildtype lung cancer cells (Figure 10) or cells overexpressing RASSF1C or PIWIL1 with piR-35127 and piR-46545 (Figure 11) decreased cell migration/invasion. The inhibitory effects of overexpressing piR-35127 and piR-46545 on lung cancer cell migration/invasion are consistent with our previously published work showing overexpression of piR-35127 and piR-46545 decreases lung cell proliferation and colony formation of breast and lung cancer cells [16]. Our current findings further suggest that piR-35127 and piR-46545 could function as anti-metastasis tools as well, and perhaps down-regulation of piR-35127 and piR-46545 might contribute to lung cell transformation and progression. We do not know if the inhibition of lung cancer cell migration/invasion by piR-35127 and piR-46545 may involve modulation of P4HA2 or PLOD2 expression. Thus, further detailed investigation of the RASSF1C-PIWIL1-piRNA pathway is needed to definitively determine its role in contributing to cell stemness and TME/ECM remodeling and its impact on breast and lung cancer metastasis. This could, in turn, lead to further understanding of how P4HA2 and PLOD2 and downstream gene networks are regulated by this novel pathway and might lead to the development of future effective lung prognostic and anti-metastatic tools such as piRNAs.

## 5. Conclusions

**Figure 12:**
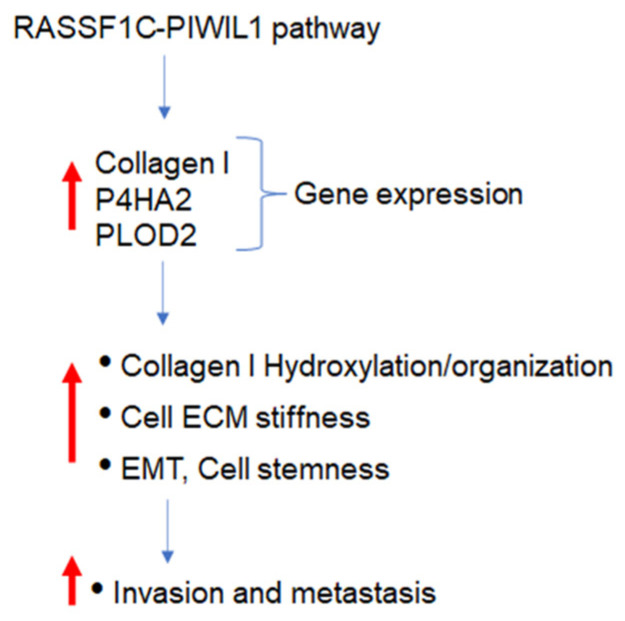
A working model of the RASSF1C-PIWIL1 pathway modulation of Collagen I, P4HA2, and PLOD2 gene expression and its impact on promoting lung TME remodeling, EMT, cell stemness, invasion, and metastasis.

These findings suggest that the RASSF1C-PIWIL1-piRNA pathway could potentially present a new dimension of regulating key genes influencing breast and lung TME remodeling and metastasis (Figure 12). However, further detailed mechanistic studies are crucial to validate this idea.

## Figures and Tables

**Table 1:** RASSF1C up-regulates P4HA2 and PLOD2 gene expression. Microarray data show that RASSF1C overexpression in breast cancer cell line T47D (T47D-1C) and in the lung cancer cell line H1299 (H1299-1C) promotes expression of P4HA2 and PLOD2 gene expression compared to controls.

Gene	Fold Change T47D-1C H1299-1C	*p*-Value
P4HA2	3.7 2	<0.01
PLOD2	1.6 1.3	<0.01

## Data Availability

Not applicable.
